# Designing Magnetism in High Entropy Oxides

**DOI:** 10.1002/advs.202200391

**Published:** 2022-02-11

**Authors:** Alessandro R. Mazza, Elizabeth Skoropata, Yogesh Sharma, Jason Lapano, Thomas W. Heitmann, Brianna L. Musico, Veerle Keppens, Zheng Gai, John W. Freeland, Timothy R. Charlton, Matthew Brahlek, Adriana Moreo, Elbio Dagotto, Thomas Z. Ward

**Affiliations:** ^1^ Materials Science and Technology Division Oak Ridge National Laboratory Oak Ridge TN 37831 USA; ^2^ Center for Integrated Nanotechnologies Los Alamos National Laboratory Los Alamos NM 87545 USA; ^3^ University of Missouri Research Reactor The University of Missouri Columbia MO 65211 USA; ^4^ Department of Materials Science and Engineering University of Tennessee Knoxville TN 37996‐4545 USA; ^5^ Center for Nanophase Materials Sciences Oak Ridge National Laboratory Oak Ridge TN 37831 USA; ^6^ Advanced Photon Source Argonne National Laboratory Lemont IL 60439 USA; ^7^ Neutron Science Division Oak Ridge National Laboratory Oak Ridge TN 37831 USA; ^8^ Department of Physics and Astronomy University of Tennessee Knoxville TN 37996 USA

**Keywords:** disorder, exchange bias, frustration, high entropy oxides, magnetism

## Abstract

In magnetic systems, spin and exchange disorder can provide access to quantum criticality, frustration, and spin dynamics, but broad tunability of these responses and a deeper understanding of strong limit disorder are lacking. Here, it is demonstrated that high entropy oxides present a previously unexplored route to designing materials in which the presence of strong local compositional disorder may be exploited to generate tunable magnetic behaviors—from macroscopically ordered states to frustration‐driven dynamic spin interactions. Single‐crystal La(Cr_0.2_Mn_0.2_Fe_0.2_Co_0.2_Ni_0.2_)O_3_ films are used as a model system hosting a magnetic sublattice with a high degree of microstate disorder in the form of site‐to‐site spin and exchange type inhomogeneity. A classical Heisenberg model simplified to represent the highest probability microstates well describes how compositionally disordered systems can paradoxically host magnetic uniformity and demonstrates a path toward continuous control over ordering types and critical temperatures. Model‐predicted materials are synthesized and found to possess an incipient quantum critical point when magnetic ordering types are designed to be in direct competition, this leads to highly controllable exchange bias behaviors previously accessible only in intentionally designed bilayer heterojunctions.

## Graphical abstract

High entropy oxides provide a previously unexplored route to designing magnetic behaviors not possible in less complex materials. Theoretical predictive models are developed and used to inform single‐crystal film synthesis to gain continuously tunable access to a full range of magnetic states—from antiferromagnetism to ferromagnetism to frustrated dynamical magnetism.

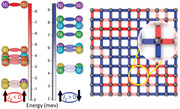

## 1 Introduction

Magnetism is an easily observable quantum phenomenon and is a cornerstone of technologies ranging from magnetic memory, to spintronics, to future quantum sensing and computing applications.^[^ ^1^, ^2^, ^3^, ^4^ ^]^ The type and strength of magnetic state in crystalline materials is dictated by discrete values, such as the number of unpaired electrons and type of magnetic exchange interactions populating the crystal lattice. The development of predictive design strategies aimed at tailoring functional magnetic responses is then entirely reliant on our ability to not only computationally forecast what parameters must be present on a lattice to generate a required magnetic behavior, but these parameters must also be translated into real materials through synthesis. This is fundamentally challenging, since direct continuously tunable control over spin (*S*) and magnetic exchange (*J*) values are needed to access a precisely defined parameter space; however, these values must be experimentally created using the limited set of spin active elements. There are several indirect methods used to influence magnetic responses in strongly correlated materials, such as where heteroepitaxial effects and defect engineering are commonly used to manipulate spin‐coupled charge and orbital parameters.^[^ ^5^, ^6^, ^7^, ^8^, ^9^ ^]^ Direct modification to the underlying *S* and *J* values using substitutional doping approaches is traditionally limited by thermodynamic processes during synthesis which can cause like elements to cluster or form secondary phases.^[^ ^10^ ^]^ Thus, while substitutional doping promises the most direct route to accessing the magnetic parameters used in computational approaches, enthalpic effects during synthesis can drive element segregation which limits mixing and reduces the number of desired composite microstates that exist in well‐mixed regions.^[^ ^11^ ^]^ Recent developments in entropy‐assisted synthesis provide a path to overcoming enthalpic effects and create well‐mixed systems.^[^ ^12^ ^]^

By greatly increasing the number of elements present in a material during synthesis, it is possible for entropic effects to dominate over enthalpy during crystal formation.^[^ ^13^, ^14^ ^]^ The governing entropy ensures exceptional mixing, which maximizes the number of local microstates hosted on a lattice.^[^ ^15^ ^]^ Increasing local disorder in entropy stabilized materials is linked to significant functional improvements in thermal transport,^[^ ^16^, ^17^ ^]^ ionic conductivity,^[^ ^18^, ^19^ ^]^ and catalytic responses^[^ ^20^, ^21^ ^]^ over less complex materials. Recent work has shown that this compositional disorder can be accommodated on ordered single crystalline lattices, which reduces the need to consider extrinsic parameters in computational models.^[^ ^22^, ^23^, ^24^, ^25^ ^]^ With the development of single‐crystal synthesis, it is possible to more rationally design these systems to address previously inaccessible fundamental questions related to disorder while disentangling intrinsic from extrinsic effects that might be present in nonsingle‐crystal form factors. In this way, the effects of known element‐specific parameters and interatomic couplings can be microscopically mapped to materials of extraordinary compositional complexity to predict macroscopic collective behavior, and critically, these compositionally complex but structurally uniform crystals can be synthesized in the real world.

Unlike high entropy alloys built from metal–metal bonded elements,^[^ ^13^, ^26^ ^]^ high entropy oxides give access to functionalities in covalent and ionic bonded materials. While the metal‐bonded high entropy alloys are limited in their range of crystal structures, stability, and accessible magnetic interaction pallet, the addition of an anion sublattice enables a broad range of stable crystal structures and greater access to functional diversity.^[^ ^14^, ^15^ ^]^ Still, considering the sensitivity of spin behavior to bond angles and cation orbital filling in many oxide systems,^[^ ^27^, ^28^ ^]^ it is surprising that high entropy oxides^[^ ^29^ ^]^ hosting high levels of compositional disorder have been reported to support signatures of long‐range magnetic ordering in both relatively simple rock salt lattices^[^ ^30^, ^31^, ^32^ ^]^ and more complicated spinel^[^ ^33^, ^34^ ^]^ and perovskite^[^ ^35^, ^36^, ^37^ ^]^ structures. In explaining this emergent long‐range magnetic order in the rock salt (Mg_0.2_Co_0.2_Ni_0.2_Cu_0.2_Zn_0.2_)O, the antiferromagnetic order is found to be a direct result of each of the species having antiferromagnetic exchange interactions.^[^ ^38^ ^]^ Of broader interests, the local exchange and spin disorder hosted in these compositionally complex systems may provide critical insights on the role of disorder in degeneracy‐driven magnetic dynamics and phase competition—where mixed phase could lead to the emergence of interesting phenomena like exchange bias. In all cases, the mechanism of ordering must revolve around the type and strength of exchange couplings populating the lattices as a function of the elemental compositions. This is fundamentally different from the itinerant RKKY or dilute‐type magnetism observed in metal–metal bonded systems.^[^ ^13^, ^39^ ^]^ The addition of the anion sublattice in the high entropy oxides promises a greater diversity of magnetic interactions enabled by localized rather than delocalized electron coordination.

The presence of correlated states in high entropy transition metal oxides and the resulting increase in complex microstates comes at the cost of greatly decreasing the feasibility of producing accurate descriptions of these systems using computationally intensive first principles approaches.^[^ ^40^ ^]^ Even with the large body of work in literature on low complexity transition metal oxide materials, functional theoretical modeling of the high entropy oxides has been extremely limited. Electron correlation and many‐body effects combined with the range of possible microstates in a compositionally complex system have made precise quantitative calculations impractical.^[^ ^41^ ^]^ For example, a *B*‐site in a simple ternary *AB*O_3_ perovskite is surrounded by like elements on the *B*‐O‐*B* sublattice so has only 1 possible nearest neighbor and next nearest neighbor arrangement. In a high entropy perovskite oxide system with five different cations residing on the *B*‐site sublattice, there are more than 200 possible nearest neighbor combinations for each of the five different elements. Importantly, the microstates with the highest probability of being populated are those in which all five elements are present in the next nearest neighbor positions. That is to say that the importance of low complexity microstates whose values can be directly harvested from literature is reduced as the number of different elements in the magnetic lattice increases.^[^ ^42^ ^]^ Thus, correlated high entropy oxides possess local environments that are fundamentally different than the less complex parent compounds which can be expected to lead to functionalities not present in low complexity systems. To gain insights into the high complexity systems, there is an outstanding need to experimentally control for cation variances and identify a means to model emergent responses in a computationally accessible manner.

In this work, a range of single‐crystal entropy‐stabilized *AB*O_3_ perovskite films is synthesized to probe the role of site‐to‐site spin and exchange interaction variances in stabilizing emergent magnetic behaviors. The complexity of the system is found to provide tunability and functionality not present in any of the ternary or half‐doped quaternary parents or as a simple sum of their properties. Neutron diffraction and magnetometry show that the compositionally disordered systems can paradoxically host long‐range magnetic order, while manipulation of the *S* and *J* parameters through cation ratio permits continuous control of magnetic phase from antiferromagnetism (AFM), to degenerate, to ferromagnetism (FM). Tuning of the coexisting magnetic phase composition is also shown to allow the design of exchange bias behaviors in monolithic single‐crystal films, which have, until now, only been observable in AFM‐FM bilayer heterojunctions or 2D layered bulk systems.^[^ ^43^ ^]^ In spite of the extraordinary levels of microstate complexity, a classical Heisenberg model populated with composite parameter states is found to produce an astonishingly accurate magnetic phase diagram, that provides insights into the mechanisms driving the emergence of macroscopic magnetic states. This provides a practical means of predicting how to manipulate the parameter ratios to stabilize desired states for functional design of materials with high compositional complexity.

## 2 Results

La(Cr_0.2_Mn_0.2_Fe_0.2_Co_0.2_Ni_0.2_)O_3_ (L5BO) is an ideal high entropy oxide system to examine the role of local magnetic disorder on the emergence of macroscopic magnetic behaviors. Structurally, this *AB*O_3_ perovskite possesses full mixing of the *B*‐site cations while maintaining long‐range single‐crystal lattice uniformity; the details of which can be found in previous works.^[^ ^25^, ^36^ ^]^ There are many theoretical and experimental studies on the parent compounds, LaCrO_3_, LaMnO_3_, LaFeO_3_, LaCoO_3_, and LaNiO_3_ and a range of interfacial and co‐doping studies that provide insights into expected spin, charge, and oxygen‐mediated coupling types between different 3d transition metal cations across a range of structural distortions and dimensionalities.^[^ ^44^, ^45^, ^46^, ^47^, ^48^, ^49^, ^50^, ^51^, ^52^, ^53^, ^54^, ^55^, ^56^, ^57^, ^58^, ^59^, ^60^, ^61^ ^]^ These studies provide a starting point to understand nearest neighbor interactions and point to there being a wide range of different spin and exchange interactions coexisting in the high entropy systems; however these previous works provide no direct insights into how magnetism might evolve when combined in a randomly populated system where next nearest neighbors can vary widely.

**Figure 1:**
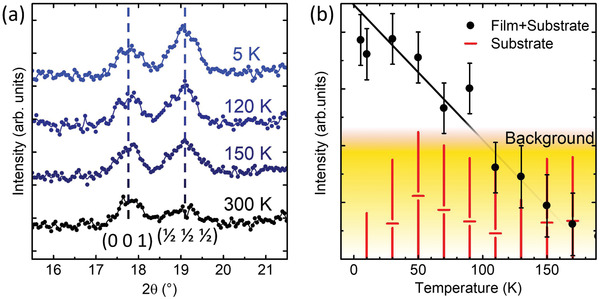
Observation of G‐type antiferromagnetic ordering in L5BO bulk and film. a) Temperature‐dependent neutron diffraction of bulk polycrystalline L5BO sample shows the (½ ½ ½) peak emerges and increases in intensity with decreasing temperature which is consistent with the onset G‐type antiferromagnetism. No other temperature‐dependent peaks are observed and the structural (0 0 1) peak is included for reference. b) Neutron diffraction data taken for the order parameter of the (½ ½ ½) peak in a L5BO single‐crystal film on an LSAT substrate show the onset of magnetic order emerging near the predicted Neel temperature.

Neutron diffraction on the L5BO system shows that this complex mix of local microstates hosts robust long‐range macroscopic AFM ordering in both the bulk powder ceramic and single‐crystal thin film forms (**Figure** **1**). These results are presented considering the cubic Miller indices of the film, where the (0 0 1) peak refers to the structural and temperature‐independent feature in the powder diffraction, and the (½ ½ ½) peak is an AFM Bragg peak occurring only below *T*_N_. The temperature dependence of the (½ ½ ½) peak of the L5BO in polycrystalline form provides an order parameter that shows an onset of AFM occurring between the measurements taken at 300 and 150 K, which is consistent with Mössbauer studies in previous studies of L5BO powder produced by spray pyrolysis.^[^ ^35^ ^]^ Since single crystals may help preclude possible extrinsic contributions related to complexity of grain size, surface effects, and inhomogenous mixing of constituents, neutron diffraction is also performed on single‐crystal films grown on near lattice matched (LaAlO_3_)_0.3_(Sr_2_TaAlO_6_)_0.7_ (LSAT) substrates to allow the film to maintain a nearly cubic structure.^[^ ^36^, ^42^ ^]^ The temperature‐dependent evolution of the (½ ½ ½) peak for a 90 nm film is shown in Figure 1b and demonstrates a clear G‐type AFM transition in the L5BO film. These single‐crystal films show no sign of relaxation and have rocking curve widths <0.08°. While the exact onset temperature is hidden at higher temperatures by the substrate background signal, the onset trend agrees with irreversibility in temperature‐dependent magnetization in the field cooled versus zero field cooled SQUID magnetometry results, implying that the Neel temperature occurs near 180 K.^[^ ^42^ ^]^

**Figure 2:**
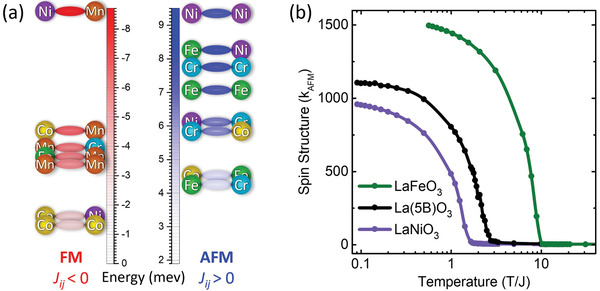
Comparison of ABO_3_ transition metal oxides’ magnetic behaviors. a) Magnetic ordering type and transition temperatures for lanthanide transition metal oxides show the wide range of functional phase space accessible by changing the B‐site cation. Red denotes ferromagnets and blue denotes antiferromagnets. b) Comparison of calculated spin structure factor *S*(*k*_AFM_) at wavevector *k*_AFM_ = (*π*, *π*, *π*) versus temperature, *T*/*J*, where *J* is taken as 82 K, for LaFeO_3_, LaNiO_3_, and entropy‐stabilized La(Cr_0.2_Mn_0.2_Fe_0.2_Co_0.2_Ni_0.2_)O_3_. The model matches known transition behaviors for the simple ternary compounds and predicts a G‐type antiferromagnetic ordering in the L5BO system with a *T*_N_ ≈ 210 K.

The observed long‐range magnetic order is remarkable considering the local spin and exchange disorder hosted within the lattice. In understanding how order emerges from disorder, a classical model for a square lattice populated by a range of possible exchange interactions distributed across the lattice can provide initial insights.^[^ ^11^, ^62^, ^63^ ^]^ A calculation of the magnetic order parameter can be found by considering the highest probability microstates in a random distribution on the perovskite lattice. The fact that the cations in the L5BO system randomly populate a lattice that, while uniform, is neither identical to a specific parent material nor an average of all parents^[^ ^25^ ^]^ makes direct gathering of *S* and *J* values from literature difficult. There is little known about the way many of these cations will couple for a single isolated bond while neglecting the other five nearest neighbors, which could influence charge and orbital state. It is important to assign values that would have the highest probability of being most valid when randomly distributed throughout a chaotic compositional landscape hosted on a well‐ordered lattice. In a randomly mixed system, the central element has the highest probability of being coordinated to several different transition metals. There are uncertainties in selecting spin and exchange values in a random *B*‐site occupancy, which results from the influence of nearest neighbors that are not present if these values were to be taken from previous reports on low complexity ternary or quaternary parent systems; further discussion of the simplified choice of spin and exchange values, summarized in **Figure** **2**a, can be found in the Supporting Information. However, there is a clear trend in exchange type preferences for two of the five elements. The overall dominance of AFM can be linked to Fe which has antiferromagnetic tendencies in four of its five oxygen mediated bonds, i.e., in Fe—O—Fe, Fe—O—Cr, Fe—O—Co, and Fe—O—Ni. Whereas, Mn strongly favors FM coupling.^[^ ^42^ ^]^

Figure 2b shows the results of the Monte Carlo simulation for the calculated magnetic responses of the L5BO system and two related and well‐studied ternary systems. Despite the many FM couplings used, no peak at the FM (0,0,0) is detected. Results are presented in Figure 2b where *S*(*k*_AFM_), with *k*_AFM_ = (*π*, *π*, *π*), is shown as a function of temperature for the pure 100% Fe—O—Fe case (with the largest spin), the 100% Ni—O—Ni (with the smallest spin), and the 20% equiatomic *B*‐site populated L5BO. Considering that *T*_N_ = 740 K for pure LaFeO_3_, this establishes the scale *J* in Figure 2b to be ≈82 K after rescaling of existing Monte Carlo results, giving a theoretical prediction that L5BO is AFM with *T*_N_ ≈ 210 K.^[^ ^64^ ^]^ The AFM found in simulation is percolated rather than isolated, which matches the long‐range AFM order observed in experiment. As only a single transition temperature is observed, it appears to be possible to grossly design critical temperature and macroscopic ordering type by selecting elemental compositions based on the average of their *S* and *J* parameters. This opens continuously tunable magnetic phase spaces that are not accessible using less complex compositions where enthalpy‐dominated synthesis results in local clustering and disruption of structural uniformity. However, the percolated AFM state in L5BO should not be confused with a traditional macroscopically phase pure ordering where all nearest neighbors have AFM exchange interactions. In the equiatomic L5BO system, the FM bonds account for 2/5 of the total exchange interactions in the material. Despite the dominance of long‐range AFM, regions of ferromagnetically coupled neighbors are clearly observed via simulation and experiment^[^ ^36^, ^37^ ^]^ embedded in a continuous antiferromagnetic matrix. These FM regions appear in simulations at the same critical temperature as AFM order but are not coherently coupled, which prevents percolation of the FM state.^[^ ^36^ ^]^ This intrinsic frustration in the L5BO magnetic lattice is highlighted by the noncollinearity of the equilibrated spins; and while AFM order dominates, a simple average of the parent oxides' magnetic state does not complete the story. The distribution of superexchanges, *J*(***S*** *_i_·* ***S*** *j*), prior to equilibrium explains how FM character may play a more visible role at higher temperatures.^[^ ^42^ ^]^

**Figure 3:**
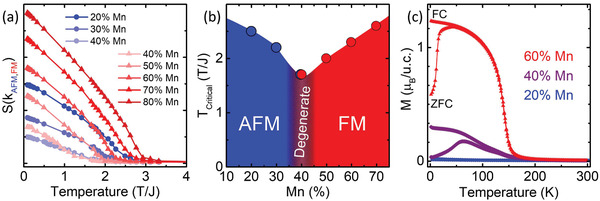
Predictive modeling and experimental validation of magnetic phase diagram as a function of increasing Mn concentration. a) Spin structure factor *S*(*k*_max_) calculated by varying the % of Mn with the other four elements equally distributed in %, where *k*_max_ is either (0,0,0) (FM, red) or (*π*, *π*, *π*) (AFM, blue) and obtained using Monte Carlo on a 10 × 10 × 10 cluster. At 40% Mn, both AFM and FM orders are percolated in the spin structure, which is why both order parameters are presented in the plot. b) The phase diagram derived from the computational model as a function of Mn content. c) Field cooled and zero field cooled temperature‐dependent magnetization taken under 1 kOe field for experimentally synthesized films.

To further test the relationship of AFM and FM in the L5BO system, the effects of iteratively shifting the composite state to lower *J* are modeled by increasing the ratio of Mn concentration in the lattice.^[^ ^42^ ^]^ Analyzing the set of superexchange values, X‐O‐Y links containing Mn favor ferromagnetism. Consequently, it is expected that increasing the relative Mn concentration in the Monte Carlo simulations should eventually lead to global ferromagnetism. Comparative Monte Carlo simulations provide expected spin structure factors as the percentage of Mn increases in relation to the other transition metals populating the lattice, where 20% is equiatomic L5BO. In **Figure** **3**a, it is shown that as % Mn initially increases to 30%, the Néel temperature decreases, but still S(0,0,0) is negligible and indicates no clear FM order. However, at 40%, an FM signal at *k*_FM_ = (0,0,0) develops and in concert with the AFM signal at *k*_AFM_ = (*π*, *π*, *π*). This is indicative of a degenerate tipping point between phase preferences, visualized as an incipient quantum critical point in Figure 3b. Both signals grow at the same temperature upon cooling. Snapshots of MC simulations visually suggest that the FM clusters have percolated at the 40% Mn concentration, with both the FM and AFM regions being coherent over long distances. At 50% Mn or larger, the AFM *S*(*k*) becomes negligible. Increasing Mn to 50% and beyond leads to the FM and AFM states switching roles, with unpercolated AFM clusters being embedded in the fully percolated FM matrix. From this, we see that the model predicts an ability to select and control the dominant percolated magnetic phase, the *T*_N_ and *T*_C_ of the percolated phases, and that it is even possible to balance the *J* and *S* composition in such a way that both AFM and FM can be fully expressed and coexisting as seen in the 40% Mn composition.

Single‐crystal films of La(Cr_0.15_Mn_0.4_Fe_0.15_Co_0.15_Ni_0.15_)O_3_ (40% Mn) and La(Cr_0.1_Mn_0.6_Fe_0.1_Co_0.1_Ni_0.1_)O_3_ (60% Mn) are synthesized to test the model's predictions experimentally.^[^ ^42^ ^]^ In Figure 3c, temperature‐dependent magnetizations of the synthesized films show that increasing Mn concentration significantly changes the magnetic responses in agreement with the model. The 60% Mn concentration presents a single sharp upturn in moment consistent with an FM *T*_C_ ≈ 160 K. The 40% Mn composition has a slightly lower critical temperature than the 20% and 60% compositions and a more gradual transition region as would be expected for competing AFM and FM phases. Precise *T*_N_ and *T*_C_ cannot be extracted from magnetometry alone in this mixed system; however, if the local spin and exchange landscape in the synthesized films does mimic the complexity predicted in the model, there should be a high degree of exchange frustration and spin disorder populating these lattices. This would lead to behaviors which may mimic those traditionally observed at the interface of thin‐film heterostructures comprised of interfacing AFM and FM layers, where intentional design of these exchange interaction discontinuities is used to manipulate exchange bias effects for spin valve, logic, and storage applications.^[^ ^65^ ^]^ Recent work has shown that increasing the disorder and uncompensated spin populations at the interface of these heterostructures may be the dominating influence in all AFM–FM exchange bias devices.^[^ ^43^ ^]^ From this, the extraordinary level of spin disorder and percolative nature of the AFM and FM phases hosted in the high entropy oxide films may provide the needed environment to generate exchange bias responses in 3D monolithic systems.

**Figure 4:**
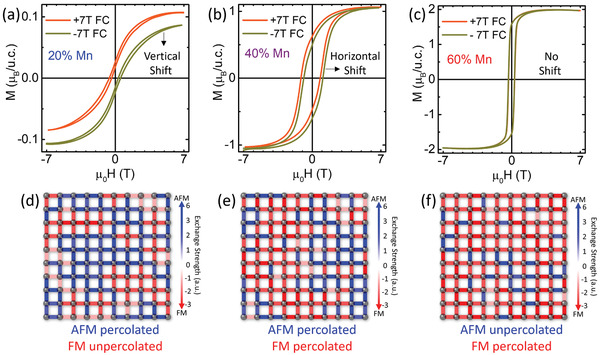
Exchange bias responses in each of the compositions with corresponding representative cross‐sections from the 10 × 10 × 10 Monte Carlo simulations. Magnetization loops taken at 2 K after field cooling under +/−7 T; a) L5BO with 20% Mn concentration shows a vertical loop offset; b) 40% Mn concentration shows a horizontal loop shift associated with traditional exchange bias response; c) 60% Mn concentration shows no measurable loop offset after field cooling. The corresponding snapshots of calculated (*J* ***S*** *_i_* · ***S*** *_j_*) cross‐sections help visualize how the local magnetic structure drives exchange bias responses, as local AFM (blue), FM (red), and near degenerate (lightly shaded) superexchange values evolve with Mn concentration. d) 20% Mn presents a fully percolated AFM state with small regions of unpercolated FM. e) 40% Mn has percolated AFM and FM phase coexistence. f) 60% Mn has percolated FM but discontinuous unpercolated AFM.

The comparative positive and negative field‐cooled magnetization loops shown in **Figure** **4**a–c indicate the presence and clear evolution of exchange bias behaviors with Mn concentration—moving from a vertical loop shift at 20% Mn, to a horizontal loop shift at 40% Mn, to a loss of biasing effects at 60% Mn. These functional changes can be understood as arising from a shift in the dominant percolative phases populating the lattice, where Figure 4d–f provides corresponding representative 10 × 10 cross‐sections from the equilibrated Monte Carlo simulations. In the 20% Mn system, the AFM phase alone is percolated. The vertical shift in magnetization results from aligning pockets of uncompensated or isolated FM moments, which creates a surplus magnetization when field cooling the sample below the AFM phase's blocking temperature.^[^ ^43^, ^66^, ^67^ ^]^ In the 40% Mn system, both the AFM and FM phases are percolated. The horizontal shift in magnetization is clear evidence of the traditional exchange biasing behavior associated with coexisting and coupled FM and AFM phases of similar energy scale.^[^ ^68^, ^69^ ^]^ In the 60% Mn system, the FM phase alone is percolated. There is no observable loop shift and the saturation moment aligns well with that expected for this composition of Mn in the *S* = 3/2 state.^[^ ^67^ ^]^

This control over magnetic coupling in 3D monolithic, single phase, single‐crystal films is remarkable, as the exchange bias response is traditionally associated with heterostructured or 2D layered magnetic materials, where direct coupling is subject to multiple crystalline components. These observations may present an important new direction in understanding the dominating mechanism of exchange bias behaviors more generally. Here, we see that manipulating the local spin disorder can be used to drive exchange bias behaviors in the monolithic single‐crystal films which resemble responses normally only accessible through intentionally designed heterojunctions; this provides important new insights into recent proposals that spin‐disorder‐driven glassiness can be a dominating factor in generating exchange bias responses.^[^ ^43^ ^]^

## 3 Discussion and Conclusions

Traditional enthalpy‐driven synthesis approaches often create materials that possess unintended secondary crystal phase formations or defects which generate extrinsic contributions when more than a few elements are combined. This limits the synthesis of desired functional states in a continuously tunable manner and excludes simple models using only intrinsic parameter variables. Experimental access to narrow regions of calculated parameter space is a critical need to enable computational materials design strategies. The presented work demonstrates that the compositionally disordered but positionally ordered lattices produced using entropy‐assisted synthesis may greatly simplify our ability to create magnetic materials by design. Beyond the continuously tunable critical temperatures of the dominant magnetic phase in films, the emergence of exchange bias responses in these single crystals is functionally important. This feature is entirely unique from the parent oxides and may lead to the development of spin‐based electronics that do not rely on heterostructuring.

Manipulating the strength and type of coexisting and randomly distributed microstates populating a well‐ordered single crystal offers opportunities to explore the effects of disorder in the strong limit, which is particularly important in a system where frustration and degeneracy may lead to unexpected or previously inaccessible phase spaces.^[^ ^70^ ^]^ Mixing elements having similar magnitude of exchange strength but opposite sign should allow intentional design of metastability, dynamic responses, and near global frustration into the lattice. As an example, magnetic frustration has been explored extensively on triangular, pyrochlore, and artificial lattices, where observation of dynamic magnetic behaviors such as spin liquids is generally attributed to degenerate ground states relying on geometric frustration.^[^ ^71^ ^]^ In magnetically complex high entropy oxides such as those described in this work, it may be possible to replace geometric frustration with exchange frustration on a square lattice by modifying the variance of exchange couplings populating the crystal.^[^ ^72^, ^73^, ^74^ ^]^ The ability to shift local variances in spin and coupling types while maintaining position symmetries also provides exciting opportunities for designing novel Griffiths phases or quantum many‐body systems with tunable critical behaviors.^[^ ^75^ ^]^ From this context, the ability to control the scale of parameter disorder hosted on a lattice may be considered as an exploitable tuning parameter for designing responses in functional materials.

## 4 Experimental Section

### Theory

For the theoretical prediction of the magnetic behavior corresponding to L5BO, a Monte Carlo study was performed on a cubic lattice using the classical Heisenberg model defined as

(1) $$H=\sum_{<\mathrm{ij}>}J_{\mathrm{ij}}S_{i}\cdot S_{j}$$

where *S_i_* are classical spins of different magnitudes depending on which transition metal element is placed at site *i*. The symbol <*ij*> refers to nearest‐neighbor (NN) sites. For the location of the spins, a random distribution based on a probability was used such that each element covered 20% of the finite clusters employed for the simulation.^[^ ^42^ ^]^

An annealing process from high temperature (i.e., slow cooling) was employed to avoid being trapped into metastable states. Simulations of different cluster size (10 × 10 × 10 and 12 × 12 × 12) were repeated and the results for critical temperatures did not change appreciably ensuring the results were not impacted by sample size. Moreover, the Monte Carlo results shown corresponded to averages over five independent random distributions of spins, but self‐averaging rendered the five results nearly identical within the accuracy needed. This comparison provided an interesting point, by suggesting it was this average which dictated the predominant order type in the film. After equilibrium, Monte Carlo was used to measure the standard spin–spin correlations < ***S*** *_i_* · ***S*** *_i_* > in real space at all distances and from them calculated the spin structure factor *S*(***k***) by Fourier transform

(2) $$S\left(k\right)=\frac{1}{N}\sum_{i,j}\left\langle S_{i}\cdot S_{j}\right\rangle e^{ik\cdot \langle r_{i}-r_{j}\rangle }$$

where **r** *_i_* is the vector position in the cubic lattice of site *i*. Among allowed momenta, the one that maximized *S*(*k*) was searched. In all simulations of the equiatomic *B*‐site perovskite using the couplings and spins discussed above, the dominant peak in *S*(*k*) was always found to be located at (*π*, *π*, *π*), i.e., in the AFM position.

### Synthesis and Characterization

Samples were prepared using pulsed laser epitaxy on 5 mm x 5 mm x 0.5 mm SrTiO_3_ (STO) and (La_0.3_Sr_0.7_)(Al_0.65_Ta_0.35_)O_3_ (LSAT) substrates. The films and ceramic targets were synthesized as described in previous work.^[^ ^36^ ^]^ In addition to equiatomic (20%) films, the 40% and 60% Mn films were synthesized using the same growth parameters with the only difference being that the pulsed laser deposition ceramic targets were of appropriate stoichiometry. All films were single phase, epitaxial, and possessed thickness uniformity. The uniformity of mixing was well established for this system and for more details the readers were pointed to these works.^[^ ^25^, ^36^ ^]^ Film thicknesses were 56 nm for 40% Mn, 58 nm for 60% Mn, and 62 nm for L5BO (20% Mn) films. The film used for neutron diffraction (grown on LSAT) was ≈90 nm. Full synthesis and characterization details of the 20% Mn films could be found in previous works focused on synthesis and structure of equiatomic B‐site populated L5BO.

The 40% Mn and 60% Mn films were synthesized after computational modeling suggested that these compositions resided at positions in the magnetic phase diagram that possessed fully percolated coexisting FM and AFM phases for 40% and single percolated FM phase for 60%. These particular compositions had never been previously reported. Anecdotally, it was found that these films grew very easily using the identical synthesis conditions to those used for the 20% Mn sample. Figure S1 in the Supporting Information gives X‐ray diffraction (XRD) and reciprocal space map (RSM) data on the 40% Mn and 60% Mn films grown on SrTiO_3_. All films were single phased, epitaxial, and possessed excellent uniformity.

Magnetization measurements, both as a function of applied field and temperature, were performed using a Quantum Design MPMS3. A linear (with magnetic field) subtraction of the diamagnetic background of the substrate was performed for all loops. Data were normalized by considering the volume of the film (using thicknesses reported above) using the perovskite unit cell based on lattice parameters found from XRD.

Neutron diffraction measurements for films were performed at the University of Missouri Research Reactor (MURR). The L5BO powder diffraction was acquired using the powder sample diffractometer, which had a wavelength of *λ* = 1.485 Å. The powder sample was loaded into a vanadium sample cell and mounted on the cold tip of a closed‐cycle refrigerator. The thin‐film diffraction was performed on the MURR triple‐axis spectrometer (TRIAX) in the elastic mode with *E_i_* =*E_f_* = 14.7 meV and collimation settings of 60’−40’−40’−40’. Pyrolytic graphite filters were used both before and after the sample to minimize higher‐order contamination from wavelength harmonics in the beam. Measurement of the film (½ ½ ½) on STO was not possible. This was due to a superstructure peak which occurred in the cubic to orthorhombic phase transition (≈110 K) in STO which overlapped with *T*_N_ in L5BO.^[^ ^76^ ^]^ While this structural component was present in LSAT there was no temperature dependence, and an increase in intensity to the onset of magnetic ordering could therefore be attributed which was established by measuring the substrate background as shown in the main text. The onset of AFM ordering from neutron diffraction matched that of the irreversibility of *M* versus *T* in Figure S2 in the Supporting Information, from which a *T*_N_ ∼ 180 K was obtained. Due to multiple reflections, LSAT had a structural and temperature‐independent intensity at the (½ ½ ½) peak position which gave rise to a background signal that is shown in Figure 1b. From this, it could be stated that there was a clear G‐type AFM transition in the L5BO film below 100 K. The exact onset temperature was hidden by the substrate background but was consistent with a higher temperature transition as observed by irreversibility in SQUID magnetometry, powder diffraction, and the Monte Carlo modeling.

## Conflict of Interest

The authors declare no conflict of interest.

## Supporting information

Supporting Information

## Data Availability

The data that support the findings of this study are available from the corresponding author upon reasonable request.
