# Supplementary material for: Designing Magnetism in High Entropy Oxides
Source: Adv Sci (Weinh). 2022 Feb 11;9(10):2200391. doi: 10.1002/advs.202200391 (PMC8981892; doi:10.1002/advs.202200391)
Supplement: Supplementary file 1 — Supporting Information [file ADVS-9-2200391-s001.pdf]

ADVANCED SCIENCE
Open Access

Supporting Information

for *Adv. Sci.*, DOI: 10.1002/advs.202200391

Designing Magnetism in High Entropy Oxides

*Alessandro R. Mazza, Elizabeth Skoropata, Yogesh Sharma, Jason Lapano, Thomas W. Heitmann, Brianna L. Musico, Veerle Keppens, Zheng Gai, John W. Freeland, Timothy R. Charlton, Matthew J. Brahlek, Adriana Moreo, Elbio Dagotto, Thomas Z. Ward**

Supporting Information

Designing Magnetism in High Entropy Oxides

Alessandro R. Mazza[1], Elizabeth Skoropata[1], Yogesh Sharma[1,2], Jason Lapano[1], Thomas W. Heitmann[3], Brianna L. Musico[4], Veerle Keppens[4], Zheng Gai[5], John W. Freeland[6], Timothy R. Charlton[7], Matthew J. Brahlek[1], Adriana Moreo[1,8], Elbio Dagotto[1,8], Thomas Z. Ward[1,]*

[1]Materials Science and Technology Division, Oak Ridge National Laboratory, Oak Ridge, Tennessee 37831, USA

[2]Center for Integrated Nanotechnologies, Los Alamos National Laboratory, Los Alamos, New Mexico 87545, USA

[3]University of Missouri Research Reactor, The University of Missouri, Columbia, Missouri 65211, USA

[4]Department of Materials Science and Engineering, University of Tennessee, Knoxville, Tennessee 37996-4545, USA

[5]Center for Nanophase Materials Sciences, Oak Ridge National Laboratory, Oak Ridge, Tennessee 37831, United States

[6]Advanced Photon Source, Argonne National Laboratory, Lemont, Illinois 60439, USA

[7]Neutron Science Division, Oak Ridge National Laboratory, Oak Ridge, Tennessee 37831, USA

[8]Department of Physics and Astronomy, University of Tennessee, Knoxville, TN 37996, USA

*email: wardtz@ornl.gov

1. Experiment

1.1 Synthesis and Characterization

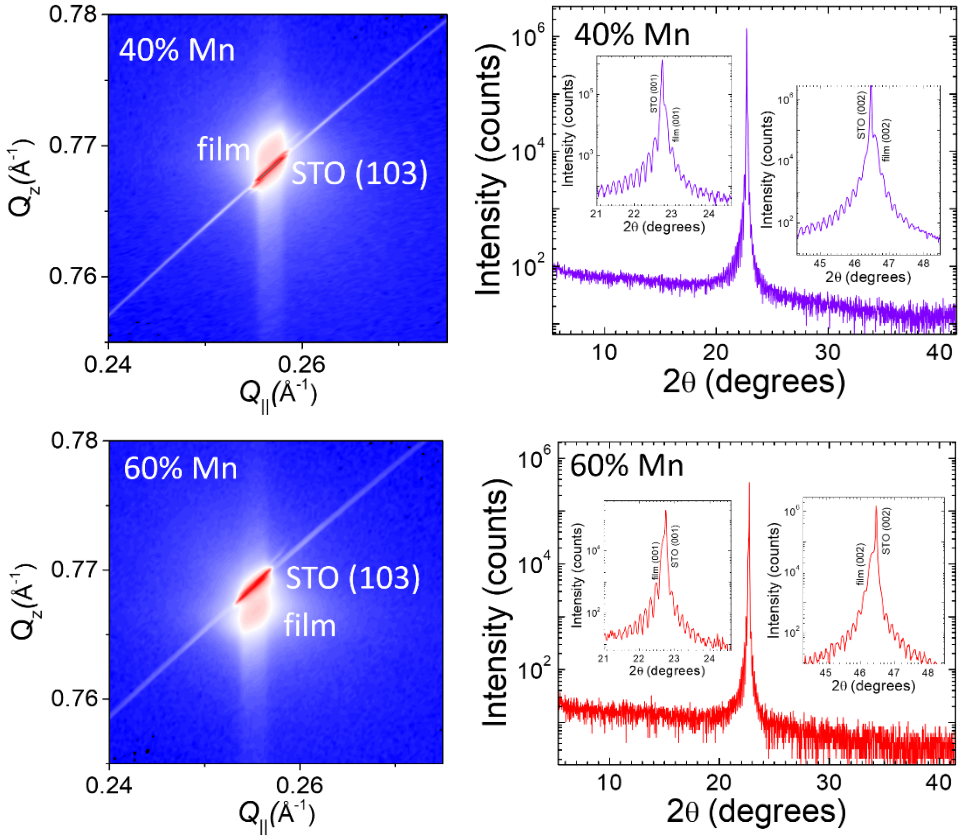

Figure S1. XRD and RSM for 40% and 60% Mn samples show excellent single phase heteroepitaxial structure. (Left) RSM around STO substrate (103) peak and (Right) *θ2θ* XRD bracketing STO (001) peak with (insets) details of (001) and (002) film peaks. (Top) is $La(Cr_{0.15}Mn_{0.4}Fe_{0.15}Co_{0.15}Ni_{0.15})O_3$ and (bottom) is $La(Cr_{0.1}Mn_{0.6}Fe_{0.1}Co_{0.1}Ni_{0.1})O_3$.

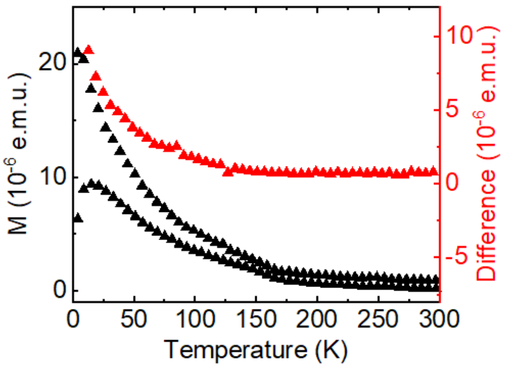

Figure S2. Magnetization vs. temperature (at 1kOe) for the L5BO film on LSAT. The onset temperature of irreversibility (red) coincides with the appearance of AFM from neutron diffraction in the text with T_N ~ 180K for LSAT.

1.2 Experiment Discussion

Pinned magnetization reported in the text was calculated by taking half the difference of the saturation moment of positive and negatively biased loops. Previous work on L5BO ceramics suggested that percolated FM regions could coexist within an AFM matrix, which could be observed as a weak exchange bias effect upon field cooling [1]. In the crystalline films, there is no apparent horizontal shift in magnetization loop which would signal exchange biasing between AFM and FM clusters. The lack of exchange bias is indicative of minimal direct coupling of these interfaces, suggesting a non-magnetic region of uncompensated spins which are influenced by the AFM blocking temperature – pinning at temperatures below ~15K. We might speculate then that disorder is key to breaking phase percolation pathways; a truly chaotic mixing of constituent elements greatly reduces the appearance of quenched disorder which could create large enough pockets to stabilize the secondary FM phase within the AFM matrix and create a direct coupling. From this, we speculate that single crystal PLD grown materials are likely to host a more uniformly mixed cation lattice due to speed of quenching during synthesis while also having fewer extrinsic structural contributions from grain boundaries and surface states than what is possible with bulk powder or ceramics forms.

The boundary between FM and AFM regions are energetically very similar, thus at higher temperatures thermal effects allow the boundary regions, believed to be composed of uncompensated spins, can contribute to the creation of a percolated FM state to arise in the slightly predominant AFM matrix. This soft boundary between phases precludes strong coupling of the percolated FM regions by the AFM matrix; thereby removing the possibility of observing a horizontal shift in field cooled magnetization loops resulting from exchange bias. At low temperature applying a magnetic field strong enough to saturate the FM regions and then field cooling to a temperature below the percolation threshold. This would effectively lock-in pockets of aligned moments (consisting of uncompensated moments surrounding AFM regions) and create a surplus magnetization that would be visible as a vertical shift in any field-dependent magnetization loops taken below the percolation onset.

2. Theory

2.1 Microstate discussion

We include a number of simplifications in the toy model used to predict the magnetic properties of the L5BO oxides. In this simplest case, we describe the assumptions we made in choosing super-exchange values and their shortcomings below. There is little known about the way many of these cations will couple for a single isolated bond while neglecting other nearest neighbors. Those neighbors can influence the charge and orbital state of the element in question.

The challenge is to assign values that would have the highest probability of being most valid when randomly distributed throughout a chaotic landscape.

We provide an example to clarify this point. In a pure ternary parent material, such as $LaMnO_3$. A central element is surrounded by 6 coordinated elements which are the same. Thus, there is only one possible state for that central element, such as Mn[Mn,Mn,Mn,Mn,Mn,Mn], where elements enclosed in the bracket are the 6 coordinated elements. However, let's assume a system where a Mn is surrounded by 5 Mn and 1 Ni. We know that the Mn will charge balance to the Ni from literature and result in $Mn^{3+} + Ni^{3+} \rightarrow Mn^{4+} + Ni^{2+}$. This would change the spin state of the Mn and make that Mn's bonding to the other 5 bonded Mn different than the idealized parent. Any changes to the local coordination system can very quickly change values that might be reported from the ideal ternary systems. Assigning exact interactions for all Mn-Mn bonds when scenarios such as this are possible will require us to make a best approximation using all available information from literature and match that to a best fit of the most widely probable populating interaction value. Consider that in the L5BO system that random probability says there is a ~75% chance that at least one of the 6 nearest neighbor transition metals surrounding the central Mn is a Ni. One could calculate the full Hamiltonian for the central Mn cation in a configuration where it was coordinated to 5 Mn and 1 Ni (let's also assume that we ignore NNN, anisotropy from distortion, etc to keep that single state calculation as simple as possible), but if one were to take this approach to modelling the whole L5BO system, it would also be necessary to calculate all 210 cation combinations that are possible around the Mn in the 6-fold coordination. This is computationally impossible. The entropy-stabilized system requires a streamlined method that captures the complexity while simplifying the minutia. We illustrate this point in **Figure S3** by showing the relative importance of the single state Mn[Mn,Mn,Mn,Mn,Mn,Mn] as a probability of its presence in the crystal. As we see, not only does this exact state have little bearing on the whole system as complexity increases, but the realistic addition of next next nearest neighbors and a crystal structure different than the bulk ternary would have a strong influence on the validity of the Mn[Mn,Mn,Mn,Mn,Mn,Mn] in the mixed systems. That is to say that a central Mn interaction with one of its coordinated Mn nearest neighbors in the state Mn[Mn,Mn,Mn,Mn,Mn,Mn] would be different than a central Mn interaction with one of its coordinated Mn nearest neighbors in the state Mn[Mn,Mn,Ni,Mn,Mn,Mn]. Below we provide details and references detailing how S and J parameters were selected for the simple Heisenberg model presented in the manuscript.

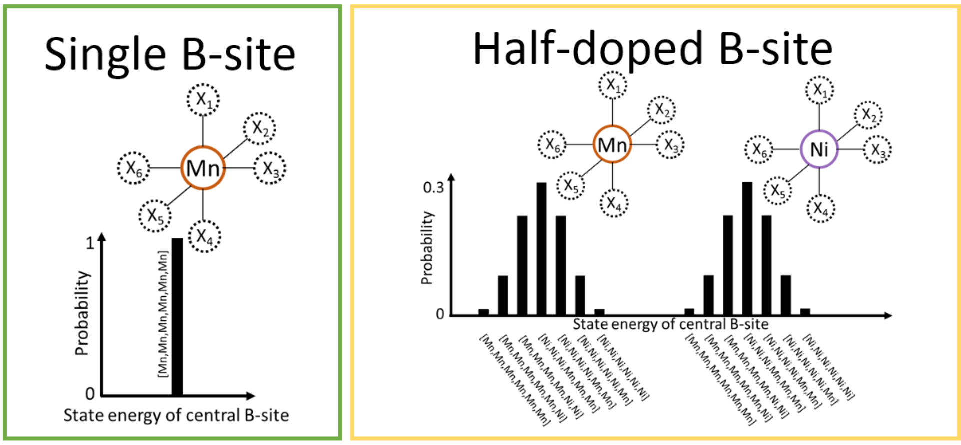

Figure S3. Illustration of how probability of state for each transition metal in the ABO_3 perovskite is strongly influenced by the number of different types of elements populating the sublattice. Note that the probability for a Mn site to be coordinated to 6 Mn nearest neighbors drops from 100%, to 1.5% in a half-doped system, to 0.0064% in the L5BO system. For high complexity materials, direct assignment of parameter values taken from less complex systems can create inconsistencies, since the probability of those microstates being present in more complex systems is reduced as complexity increases.

2.2 Selection of spin and super-exchange values

In this section, we outline the procedure we followed to obtain the superexchange values J(X,Y) used in the Heisenberg model simulation, for each of the 15 independent combinations X-O-Y. Here X and Y are any of the five elements Cr, Mn, Fe, Co, or Ni, and O represents the oxygen at each bond. First, let us recall that the classical Monte Carlo simulation of the 3D Heisenberg model defined in the main text has an antiferromagnetic Néel critical temperature T_N = 1.44 J when the spins at every site have magnitude S=1 [2]. Note also that in a bipartite lattice, as the cubic lattice used here, we can change the sign of the spins at the "even" sites, and the model transforms into the ferromagnetic classical Heisenberg model with the same Curie temperature T_C = 1.44 J. Thus, at the classical level the 1.44 is common to FM and AFM states.

The spin values are relatively easy to predict in magnitude once charge rebalancing is considered, but the values J_{ij} are more difficult because there are 15 possible combinations X-O-Y (with X,Y = Cr, Mn, Fe, Co, Ni). For the 5 X-O-X cases, this task is simplified, because the critical temperatures of the $LaXO_3$ materials can be directly related to their superexchange J, positive or negative depending on whether the order is AFM or FM. For the other 10 X-O-Y (X≠Y) cases, finding J_{ij} is more challenging and critical temperatures for 50% mixes $LaX_{0.5}Y_{0.5}O_3$, superlattices $LaXO_3$-$LaYO_3$, or in some cases interpolations between existing data are used. The summary of this effort is the following. There are 8 AFM $J_{ij}>0$ and 7 FM $J_{ij}<0$ commensurate with Figure 2, suggesting a fine balance between the two tendencies.

However, the actual magnitudes of the AFM set $J_{ij}>0$ are considerably stronger than those with FM character. Moreover, as explained above, the largest spin S=5/2 for Fe^{3+} plays an important role for the AFM dominance. Charge redistribution and disproportionation can occur when linking certain transition metals through an oxygen bond which changes accessible charge state and resulting spin state; the most well-known being the strong preference of Mn^{3+} + Ni^{3+} → Mn^{4+} + Ni^{2+} and the slightly less favorable Mn^{3+} + Co^{3+} → Mn^{4+} + Co^{2+}. This charge rebalancing is a known process in L5BO crystals as a driving factor in generating its unexpected crystalline uniformity and must be considered when selecting parameter values that have the highest probability of being in the majority[3]. With these considerations, the magnitudes of the spin of each transition metal ion are S=5/2 for Fe, S=2 for Co, S=3/2 for Mn and Cr, and S=1 for Ni[4].

As for J, let us start with the simplest 5 cases, i.e. those of the "diagonal" form X-O-X. **(i)** For X=Fe, $LaFeO_3$ is known to be a G-type AFM with T_N = 740 K [5]. By a simple rescaling of parameters, namely introducing the S=5/2 of Fe, we arrive to the equality 740 K = 1.44 J(Fe,Fe) (5/2)(5/2), leading to **J(Fe,Fe) = +82 K** which was used extensively in the main text.
(ii) For X=Cr, $LaCrO_3$ is also known to be a G-type AFM but with T_N = 290 K [6]. By the same procedure as in (i) but using S=3/2, we obtain **J(Cr,Cr) = +90 K**, similar to J(Fe,Fe).
For the next three cases, the experimental information is harder to judge.
(iii) For X=Mn, $LaMnO_3$ is known to be an A-type AFM with T_N = 140 K [7]. This magnetic arrangement has wavevector (0,0,π), i.e. FM in plane and AFM between planes. However, under strain the entire system becomes FM [8] showing that the FM and A-type AFM states are close in energy. Employing a direction-dependent J(Mn,Mn) would add too much complexity to the theory description, thus for simplicity in our study we will use a FM superexchange (as our films are strained) in all three directions. Employing the same strategy as before in (i) leads to **J(Mn,Mn) = -40 K** using S=3/2. S=3/2 is also practically reasonable, as charge redistribution is a well-known occurrence when Mn has a nearest neighbor of other transition metals such as Ni or Co[9,10], which is required in the well mixed L5BO crystals. Note that in Mn-oxide compounds double-exchange physics likely dominates, with coexisting itinerant and mobile holes, thus a negative FM superexchange is merely a simplified effective description of a far more complex mechanism for ferromagnetism.
(iv) For X=Co, $LaCoO_3$ is considered to be non-magnetic due a close energy competition between the high S=2 and low S=0 spin states, caused by competing Hund interaction and crystal field split energies between the x^2-y^2 and $3z^2$-r^2 orbitals [11]. However, in thin-films $LaCoO_3$ becomes FM with T_C = 90 K [12]. As in (i), we arrive to **J(Co,Co) = -16 K** for the case

S=2. This small value of J(Co,Co) is probably a consequence of the still present competition S=0 vs 2 in the thin films. If the average Co spin were e.g. S=1, then J(Co,Co) would increase by a factor 4 to values similar to those of previous cases. Note that we used S=0 for Co as well, and thus J(Co,Y) was effectively 0. In this case, the AFM critical temperature of cobalt S=0 was found to be smaller than S=2 in L5BO.

(v) For X=Ni, $LaNiO_3$ presents a similar difficulty as $LaCoO_3$. In bulk form $LaNiO_3$ is non-magnetic in the long-range sense. However, Ni ion likely has a nonzero spin. Moreover, in superlattices a noncollinear canting state with T_N = 157 K [13] was found. Describing non-collinear spin arrangements would require antisymmetric spin-spin terms or high frustration, complicating the description. Thus, for simplicity we assume the canonical S=1 for Ni, and an AFM NN superexchange (no antisymmetric extra term). S=1 is also practically reasonable as charge disproportination is a well-known occurrence when Ni has a nearest neighbor of other transition metals such as Mn[9], which is required in the well mixed L5BO crystals. By the procedure in (i) we obtain **J(Ni,Ni)= +109 K**, comparable to Fe and Cr.

The remaining 10 non-diagonal J(X,Y) (with X≠Y) are even more complicated to find, and we will need to make some educated guesses.

(vi) For X=Fe and Y=Mn, superlattices of $LaFeO_3$ and $LaMnO_3$ indicate ferromagnetic order at T_C = 230 K [14]. Following the procedure in (i) leads to **J(Fe-Mn)=-43 K.** Note that the alloy $LaFe_{0.5}Mn_{0.5}O_3$ is also ferromagnetic albeit with T_C = 380 K [15], providing reassurance that J(Fe,Mn) is FM. Because of caveats in about charge disproportionation and structural metastability between orthorhombic and rhombohedral forms, the results in Ref [14] are here used.

(vii) For X=Fe and Y=Cr, the alloy $LaFe_{0.5}Cr_{0.5}O_3$ was reported to be AFM with T_N = 265 K [16]. This leads to **J(Fe,Cr) = +49 K**.

(viii) For X=Mn and Y=Ni, two different FM transitions were observed in alloy $LaMn_{0.5}Ni_{0.5}O_3$ at T_C = 150 K and T_C = 280 K. [17] There is a well-known charge disproportionation that occurs in this combination. For simplicity, the average was considered, leading to **J(Mn,Ni) = - 100 K.**

(ix) For X=Mn and Y=Co, for the alloy $LaMn_{0.5}Co_{0.5}O_3$ a FM transition at T_C = 230 K was reported leading to **J(Mn,Co) = -53 K**. [10]

(x) For X=Co and Y=Ni, for the alloy $LaCo_{0.5}Ni_{0.5}O_3$ a FM transition at T_C = 53 K was reported leading to **J(Co,Ni) = -18 K**. [18]

(xi) For X=Fe and Y=Co, studies of $LaFe_{0.5}Co_{0.5}O_3$ report a canted AFM state with T_N = 370 K, leading to **J(Fe,Co)=+51 K.**[19]

(xii) For X=Co and Y=Cr, studies of $LaCo_{0.5}Cr_{0.5}O_3$ report a canted AFM with T_N = 295 K, leading to **J(Co,Cr)=+68 K**.[20]

Finally, for the last three cases further assumptions must be made.

(xiii) For X=Fe and Y=Ni, studies in Ref [21] suggest low temperature glassy behavior for the 50-50 alloy which does not allow selection of a discrete value for our purposes. Thus, from the above calculated J(Fe,Fe) and J(Ni,Ni), both AFM and similar in value, we simply make an average leading to **J(Fe,Ni)=+96 K.**

(xiv) For X=Mn and Y=Cr, we could not find information about the 50-50 alloy. The only information available is either entirely theoretical or in only small doping concentrations [22,23]. However, there is a clear smooth behavior for the values of J(Mn,Y) deduced thus far (all FM): J(Mn,Mn)=-40.1 K, J(Mn,Co)=-53.2K, J(Mn,Fe)=-42.6K, and J(Mn,Ni)=-69.4K. Since Cr is closer to (Mn,Fe,Co) than Ni in the periodic table, we use the first three for an average and predict **J(Mn,Cr)=-45 K**.

(xv) For X=Cr and Y=Ni, again we did not find information for the 50-50 alloy. The above estimated FM value for J(Mn,Cr) is believed to be negative primarily because of the influence of Mn, that has all the links FM. Then, instead we consider a crude average of the other existing AFM superexchange J(Cr,Y), with Y=Cr, Fe, and Co, leading to **J(Cr,Ni)=+70 K.** The AFM character assumption is reasonable because both J(Cr,Cr) and J(Ni,Ni) are both AFM.

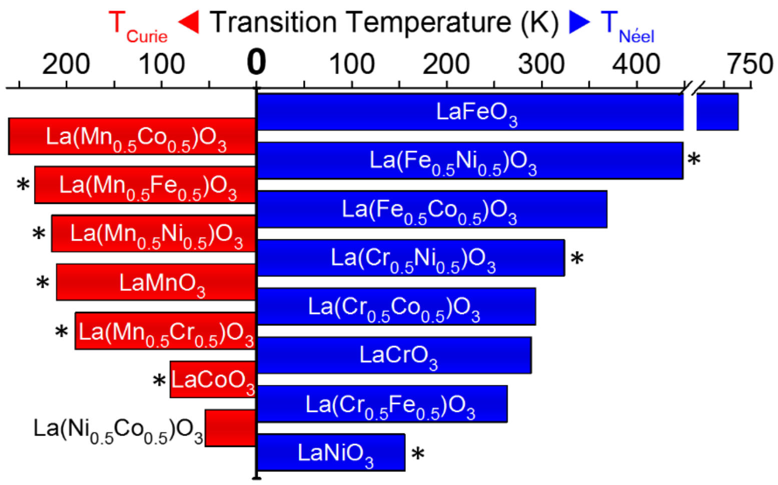

Figure S4. Extrapolated transition temperatures for La*B*O_3 and La$B_{0.5}B'_{0.5}O_3$ compounds for values and assumptions described in the selection process, where red denotes ferromagnets and blue denotes antiferromagnets. * indicates compositions that are either not thermodynamically stable in bulk or have conflicting reports of transition temperature or ordering type depending on specifics of dimensionality, charge compensation, or strain; the values are selected based on likelihood of presence within the equiatomic L5BO system.

Figure S4 provides examples observed and estimated magnetic ordering types and transition temperatures for ternary and quaternary lanthanide transition metal oxides

considering only the values taken above. Even in these low compositionally complex systems, there are often conflicting reports of different transition temperatures or type of magnetic ordering depending on crystalline quality and uniformity of structural distortions; still, it is possible to make informed decisions as to which of these reports are most relevant to a compositionally well-mixed and crystallographically uniform single crystal high entropy oxide, such as the L5BO system. Of the 15 materials, 8 are reported or expected to undergo AFM transitions with Neel temperatures within the range of 740 K to 160 K, while the remaining 7 have FM transitions with Curie temperatures within the range of 230 K to 50 K. From this, it is possible to extrapolate the local (first nearest-neighbor) exchange interactions and energies that drive the macroscopic magnetic responses observed in each of these relatively simple parent materials.

An example of a 3D rendering of a typical randomly populated 10x10x10 matrix of the resulting AFM/FM bond distribution in L5BO is given in **Figure S5**.

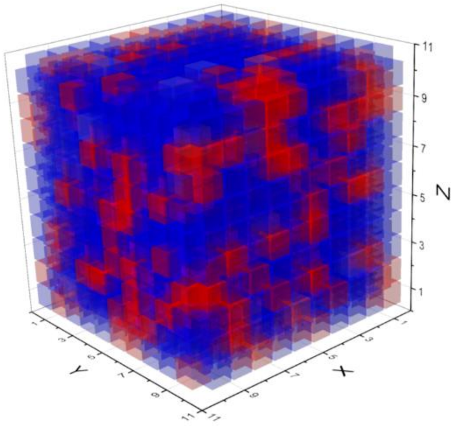

Figure S5. Example of 3D calculated (J $\mathbf{S}_i \cdot \mathbf{S}_j$) 10x10x10 cluster from which 2D slices in main text were taken. Blue(red) regions denote voxels with AFM(FM) superexchange values.

2.3 Analysis of estimated superexchange values

The crude estimations of J(X,Y) provided above seem somewhat chaotic. However, upon further scrutiny, patterns emerge. To start with, note that the signs are approximately evenly divided with 8 values AFM and 7 FM. Moreover, the largest AFM J(Ni,Ni)=+109K and the largest FM J(Mn,Ni)=-100K are quite similar in magnitude, and very close to the J(Fe,Fe)= +82K used in the main text for the critical temperature estimations.

Defining an overall scale "J" crudely in the range from 70 to 100K, the 15 superexchanges can be divided in 5 groups. Group 1 is made of J(Fe,Fe), J(Cr,Cr), J(Ni,Ni), J(Fe,Ni) and J(Cr,Co) that have similar values from the analysis above and similar to the

effective +J. Group 2 contains J(Fe,Cr) and J(Fe,Co) and its magnitude is +J/2. Group 3 contains J(Co,Co) and J(Co,Ni) and its magnitude is –J/2. Group 3 contains J(Co,Co) and J(Co,Ni) with value –J/4 (the smallest in magnitude). Group 4 contains J(Mn,Mn), J(Fe,Mn), J(Co,Mn) and J(Cr,Mn) with value –J/2. Finally, Group 5 only has J(Mn,Ni) with value –J.

Given the large uncertainties in the individual estimations of J(X,Y), forming these groups does not add more uncertainty but allow us to use only one overall scale J for the plotting of data, as shown in the main text. Moreover, this also allows us to obtain an average J, which results to be +0.23J. From this very crude extra analysis and the scale J>0, we would have predicted that L5BO of our focus should be AFM, as indeed was shown to be by the Monte Carlo analysis and even by experiments. Moreover, using for J the value J(Fe,Fe) described above, with the critical temperature 740 K, would lead to a L5BO critical temperature 0.23x740K = 170K amazingly close to experimental values. The Monte Carlo simulation presented in Figure 2b gives 210K for L5BO. Analyzing all these different lines of reasoning, and showing they are consistent with one another, provides support to our predictions.

References (all references listed here are also cited in the main text)

[1] R. Witte, A. Sarkar, R. Kruk, B. Eggert, R. A. Brand, H. Wende, H. Hahn, *Phys. Rev. Materials* **2019**, *3*, 034406.
[2] P. Peczak, A. M. Ferrenberg, D. P. Landau, *Phys. Rev. B* **1991**, *43*, 6087.
[3] M. Brahlek, A. R. Mazza, K. C. Pitike, E. Skoropata, J. Lapano, G. Eres, V. R. Cooper, T. Z. Ward, *Phys. Rev. Materials* **2020**, *4*, 054407.
[4] Materials, methods, and additional text are available as supplementary materials, **n.d.**
[5] M. Eibschütz, S. Shtrikman, D. Treves, *Phys. Rev.* **1967**, *156*, 562.
[6] B. Tiwari, A. Dixit, R. Naik, G. Lawes, M. S. Ramachandra Rao, *Appl. Phys. Lett.* **2013**, *103*, 152906.
[7] Y. Murakami, J. P. Hill, D. Gibbs, M. Blume, I. Koyama, M. Tanaka, H. Kawata, T. Arima, Y. Tokura, K. Hirota, Y. Endoh, *Phys. Rev. Lett.* **1998**, *81*, 582.
[8] J. Roqueta, A. Pomar, L. Balcells, C. Frontera, S. Valencia, R. Abrudan, B. Bozzo, Z. Konstantinović, J. Santiso, B. Martínez, *Crystal Growth & Design* **2015**, *15*, 5332.
[9] M. Kitamura, M. Kobayashi, E. Sakai, M. Minohara, R. Yukawa, D. Shiga, K. Amemiya, Y. Nonaka, G. Shibata, A. Fujimori, H. Fujioka, K. Horiba, H. Kumigashira, *Phys. Rev. B* **2019**, *100*, 245132.
[10] T. Burnus, Z. Hu, H. H. Hsieh, V. L. J. Joly, P. A. Joy, M. W. Haverkort, H. Wu, A. Tanaka, H.-J. Lin, C. T. Chen, L. H. Tjeng, *Phys. Rev. B* **2008**, *77*, 125124.
[11] M. A. Korotin, S. Yu. Ezhov, I. V. Solovyev, V. I. Anisimov, D. I. Khomskii, G. A. Sawatzky, *Phys. Rev. B* **1996**, *54*, 5309.
[12] D. Meng, H. Guo, Z. Cui, C. Ma, J. Zhao, J. Lu, H. Xu, Z. Wang, X. Hu, Z. Fu, R. Peng, J. Guo, X. Zhai, G. J. Brown, R. Knize, Y. Lu, *Proc Natl Acad Sci USA* **2018**, *115*, 2873.
[13] H. Guo, Z. W. Li, L. Zhao, Z. Hu, C. F. Chang, C.-Y. Kuo, W. Schmidt, A. Piovano, T. W. Pi, O. Sobolev, D. I. Khomskii, L. H. Tjeng, A. C. Komarek, *Nat Commun* **2018**, *9*, 43.

[14] K. Ueda, H. Tabata, T. Kawai, *Phys. Rev. B* **1999**, *60*, R12561.
[15] K. Ueda, Y. Muraoka, H. Tabata, T. Kawai, *Appl. Phys. Lett.* **2001**, *78*, 512.
[16] A. K. Azad, A. Mellergård, S.-G. Eriksson, S. A. Ivanov, S. M. Yunus, F. Lindberg, G. Svensson, R. Mathieu, *Materials Research Bulletin* **2005**, *40*, 1633.
[17] V. L. J. Joly, P. A. Joy, S. K. Date, C. S. Gopinath, *Phys. Rev. B* **2002**, *65*, 184416.
[18] T. Kyômen, R. Yamazaki, M. Itoh, *Phys. Rev. B* **2003**, *68*, 104416.
[19] V. Solanki, S. Das, S. Kumar, Md. M. Seikh, B. Raveau, A. K. Kundu, *J Sol-Gel Sci Technol* **2017**, *82*, 536.
[20] V. Solanki, Md. Motin Seikh, A. K. Kundu, *Journal of Magnetism and Magnetic Materials* **2019**, *469*, 95.
[21] M. Gateshki, L. Suescun, S. Kolesnik, J. Mais, K. Świerczek, S. Short, B. Dabrowski, *Journal of Solid State Chemistry* **2008**, *181*, 1833.
[22] D. V. Karpinsky, I. O. Troyanchuk, V. V. Sikolenko, *J. Phys.: Condens. Matter* **2007**, *19*, 036220.
[23] J. Wang, X. Hao, Y. Xu, Z. Li, N. Zu, Z. Wu, F. Gao, *RSC Adv.* **2015**, *5*, 50913.
